# Pulmonary Aspergillosis Mimicking Metastatic RCC: A Postnephrectomy Diagnostic Consideration

**DOI:** 10.1155/carm/9920083

**Published:** 2025-09-05

**Authors:** Supriya Peshin, Ramsha Sohail, Faizan Bashir, Donovan Mabe

**Affiliations:** ^1^Department of Internal Medicine, Norton Community Hospital, Norton, Virginia, USA; ^2^School of Medicine, Shiraz University of Medical Sciences, Shiraz, Iran; ^3^Shiraz Nephro-Urology Research Centre, Shiraz University of Medical Sciences, Shiraz, Iran; ^4^Department of Pulmonary Critical Care, Norton Community Hospital, Norton, Virginia, USA

**Keywords:** *Aspergillus fumigates*, mRCC, nephrology surveillance, opportunistic infection, pulmonary aspergillosis, renal cell carcinoma

## Abstract

Survivors of renal cell carcinoma (RCC), especially following nephrectomy, require long follow-up for recurrence or systemic complications. While those with a history of RCC develop pulmonary nodules often with concern for metastasis, we must consider infectious diseases, especially in patients with environmental exposures and immune alterations related to metabolic changes secondary to nephrectomy. We report a 49-year-old male with a history of RCC status postnephrectomy, history of long-term smoking, and history of significant coal dust exposure, who developed progressive pulmonary nodules. Although initially suspected to be metastatic RCC (mRCC), serial imaging demonstrated nodule enlargement without metabolic activity on PET scan, requiring further evaluation. Given the persistent respiratory symptoms, he underwent bronchoscopy with microbiologic analysis which identified *Aspergillus fumigates* and was ultimately diagnosed with chronic pulmonary aspergillosis. Our patient was successfully treated with voriconazole and over time demonstrated significant clinical improvement. In this case, we have made the observation of the diagnostic dilemma presented by the pulmonary nodules in RCC survivors and the importance of a broad differential to avoid misdiagnosis. Immune changes following nephrectomy, possible accompanying chronic kidney disease (CKD), or prolonged oncologic surveillance could place patients at risk for opportunistic infections. In order to ensure timely detection and treatment of infections that may mimic tumor progression, clinicians treating RCC survivors should integrate microbiologic diagnostics into routine pulmonary evaluations.

## 1. Introduction

Pulmonary nodules are common and their evaluation is challenging, especially in patients with a prior malignancy or history of smoking or occupational exposures [1, 2]. In renal cell carcinoma (RCC) patients, new or enlarging pulmonary nodules understandably cause concern for metastatic disease given RCC's high metastatic potential and reportedly high lung metastasis rate (up to 60%) [3, 4]. The most frequent radiographic manifestations are solitary or multiple lung nodules [4]. However, a broad differential diagnosis is essential, as nonmalignant conditions including infectious etiologies can occasionally mimic cancer progression [5].

This is especially relevant in RCC patients, where the malignancy exerts immunosuppressive effects, and nephrectomy is believed to partially mitigate these effects [6].

Fungal lung infections, most notably *Aspergillus fumigatus*, should be considered in individuals with existing structural lung disease, extended immunosuppression, or heavy environmental contacts [7, 8]. Pulmonary aspergillosis incorporates a range of clinical entities from colonization and allergic bronchopulmonary aspergillosis (ABPA) to invasive pulmonary aspergillosis (IPA) and chronic pulmonary aspergillosis (CPA) [7, 9]. CPA is frequently seen in patients with pulmonary comorbidities like chronic obstructive pulmonary disease (COPD), sarcoidosis, and tuberculosis (TB) [10].

In this case, the continued presence and progression of pulmonary nodules with right middle lobe atelectasis required thorough investigation—including bronchoscopy with microbiologic evaluation. The differential diagnosis included metastatic RCC, pulmonary carcinoid tumor, and tracheobronchial papillomatosis, given the patient's oncologic history and smoking exposure. However, the absence of positron emission tomography (PET) avidity in the nodules despite their progression raised clinical suspicion for a nonmalignant etiology [11].

Microbiologic confirmation of *A. fumigatus* on bronchoscopy highlights the key importance of early diagnostic sampling in the persistently abnormal lung. Culture-based identification is the backbone of definitive diagnosis [12, 13]. The patient was treated successfully with voriconazole, emphasizing that delays in antifungal treatment can lead to irreversible damage of the lungs [8, 14]. Clinicians caring for postnephrectomy RCC survivors should be vigilant for opportunistic infections that can mimic malignancy on imaging.

We report the case of a 49-year-old male with a history of RCC status postnephrectomy, chronic tobacco use, and prolonged coal mine exposure, who developed progressively enlarging pulmonary nodules. Initially presumed to represent metastatic RCC, the nodules were ultimately diagnosed as pulmonary aspergillosis.

## 2. Case Presentation

A 49-year-old man with a history of left RCC, status postnephrectomy, presented with worsening exertional shortness of breath, intermittent wheezing, and productive cough. He had a medical history of significant smoking history (40 pack years) and 20 years of oral coal mine exposure. There was concern for pulmonary pathology in light of his history. He did not have any prior history of TB, exposure to asbestos, COPD, or major family history of lung cancer. Therefore, with oncologic history and numerous long-standing environmental exposures, further evaluation was warranted.

Multiple enlarging pulmonary nodules, most noticeable along the right cardiac margin, were discovered on a chest CT scan (10/28/2020). This prompted a PET scan (11/02/2020) to determine whether the nodules were malignant. Surprisingly, PET imaging showed no metabolic uptake, reducing suspicion for RCC metastases. The only notable finding was stable right middle lobe atelectasis. Given the absence of metabolic activity, a conservative approach with serial imaging was initially adopted.

**Figure 1:**
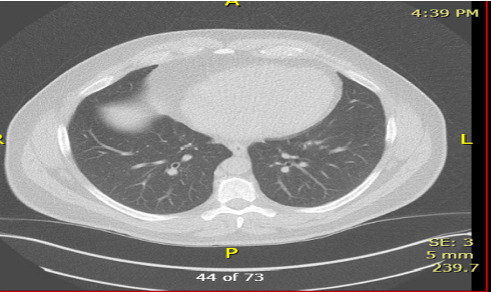
Chest CT scan findings—right lower lobe: enlargement of a previously identified nodule (now measuring 11 mm). Left upper lobe and right upper lobe: stable nodules (unchanged in size compared with prior imaging).

Following this, over 3 years, the patient underwent regular imaging to monitor nodule stability and progression:

• CT Chest (02/09/2021): stable bilateral pulmonary nodules with persistent right middle lobe atelectasis.
• CT Chest (05/05/2021): persistent noncalcified pulmonary nodules accompanied by chronic atelectasis, raising suspicion for a potential endobronchial lesion.
• CT Chest (11/17/2021): stable bilateral subcentimeter pulmonary nodules, likely benign but warranting ongoing surveillance.
• CT Chest (06/2022): no significant changes in pulmonary nodules or atelectasis.
• Low-Dose CT (07/2023): enlargement of the right lower lobe nodule (11 mm), while the nodules in the left upper and right upper lobes remain stable (Figure 1).

**Figure 2:**
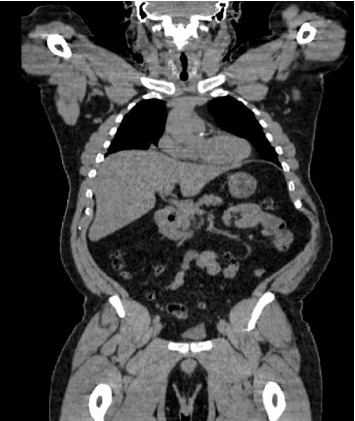
CT scan findings. Coronal contrast-enhanced CT scan of the chest showing multiple bilateral pulmonary nodules of varying sizes. Findings initially raised suspicion for metastatic disease in a patient with a history of renal cell carcinoma.

**Figure 3:**
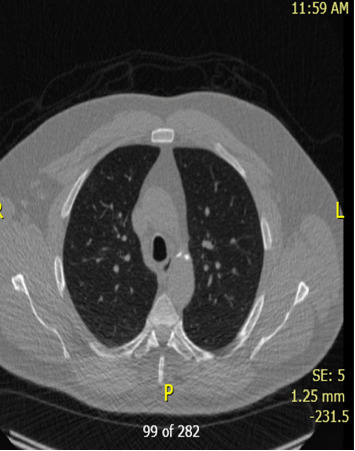
Chest CT findings. CT image of the chest showing volume loss in the right middle lobe, with fissural displacement suggestive of persistent atelectasis. These findings raise concern for possible endobronchial obstruction.

Chest CT findings, as shown in Figures 2 and 3, reveal numerous solid nodules throughout both lungs.

### 2.1. PET Scan (08/2023)

• Progressive enlargement of multiple pulmonary nodules without notable metabolic activity.
• Persistent atelectasis of the right middle lobe, likely associated with an endobronchial component.

These findings raised concern for a nonmalignant yet progressive process.

With the patient's history of RCC, smoking, and chronic inhalational exposure, the main differential diagnoses were as follows:

1. Metastatic RCC: due to the nodule advancement, metastases were a leading concern regardless of the lack of PET avidity.
2. Pulmonary carcinoid tumors: probable explanation for endobronchial involvement and progressive nodularity.
3. Tracheobronchial papillomatosis: because of chronic atelectasis and airway involvement.

Despite the combination of progressive nodules with PET negativity, additional workup of infectious etiologies was warranted, and given the patient's environmental exposure and chronic smoking history, an infectious etiology was suspected. Subsequently, bronchoscopy was performed (09/2023), which revealed (a) no signs of malignancy in cytology, (b) positive *A. fumigatus* culture from BAL, and (c) negative acid-fast bacilli (AFB) and fungal culture growth for other organisms. These results validated CPA over malignancy as the etiology of the patient's pulmonary nodules.

After the diagnosis of the pulmonary *A. fumigatus* infection, the patient was started on voriconazole, the first-line antifungal for CPA. With the patient's postnephrectomy status, renal function was monitored closely to make sure that his dosing was appropriate and there was no toxicity. He had clinical improvement and his respiratory symptoms resolved. Serial imaging follow-up was recommended to assess treatment response and ensure the nodules remained stable.

This case underscores the importance of staying alert to infections that can mimic cancer in RCC survivors. In postnephrectomy patients, immune changes may raise the risk for fungal disease, making a broad, thoughtful workup essential.

## 3. Discussion

Pulmonary nodules in patients following RCC diagnosis or treatment frequently present a diagnostic dilemma, which often makes radiologists wary of metastatic disease. However, nephrologists following up on patients postnephrectomy need to consider the possibility of infectious mimics especially if they have risk factors, e.g., chronic tobacco use or environmental exposures. While at first malignancy (given the propensity of RCC to metastasize) was the working diagnosis for this patient, the absence of substantial PET uptake along with nodule growth, led to further management and diagnostic efforts. This saw discovery of pulmonary aspergillosis.

The lungs are a common site of RCC metastasis, emphasizing the tumor's high metastatic potential [3, 15, 16]. Pulmonary recurrence has been reported up to a rate of 93% after nephrectomy, with documented cases occurring even decades later; for example, Singh et al. [17] described lung metastasis 16 years after nephrectomy. Thus, any new pulmonary lesion in an RCC survivor warrants thorough investigation.

Infectious causes including granulomatous mycobacterial and fungal infections are very well-known mimics of malignancy in the lung. These lesions can expand over time, have spiculated margins, and exhibit fluorodeoxyglucose (FDG) uptake on PET imaging, all of which can mimic an advanced metastasis. The chronic necrotizing aspergillosis, especially its solid form, can especially mislead patients at risk (e.g., with emphysema and/or tobacco smoking). There is a reasonable amount of radiologic overlap with malignancy, but diagnosis is centrally made with bronchoscopic or CT-guided biopsy [18]. In our case, bronchoscopy identified *A. fumigatus*, thereby avoiding more invasive interventions.

IPA generally occurs in immunocompromised patients or patients receiving immunosuppressive treatment. Iijima et al. [19] reported a similar case in a 67-year-old RCC survivor, originally misdiagnosed with pneumonia, whose condition improved after antifungal treatment with voriconazole. Sahlen et al. [20] described a different RCC survivor with chronic kidney disease (CKD), who was eventually diagnosed with aspergillosis after presenting with progressive dyspnea and productive cough. These cases underscore the importance of a low threshold for considering infectious etiologies in this population.

Nephrectomy in the context of RCC patients in particular is accompanied by systemic immune modulation. RCC itself inhibits immune surveillance by disrupting efficient immune cell function and encouraging immunosuppressive molecular pathways [21]. In addition, reduced renal mass may be associated with lower erythropoietin production, altered immune cell function, and a tendency toward a proinflammatory state. If CKD is also present, this immune dysfunction is compounded [22]. Uremic toxins and metabolic derangements can impair neutrophil function [23], rendering patients more susceptible to infections that may radiologically mimic malignancy.

In this case, the chronicity of the nodules, PET nonavid disease and relevant environmental exposures all suggested an infectious rather than malignant cause. This justifies a wide differential diagnosis when reviewing pulmonary findings for RCC survivors.

Given the high burden of pulmonary metastases in RCC, close collaboration between nephrology and oncology teams is essential. However, clinicians must also recognize and evaluate alternative pulmonary pathologies, especially in patients with CKD, prior smoking history, or occupational exposures. To aid in clinical decision-making, a structured diagnostic framework was applied in this case, progressing from PET imaging to microbiologic confirmation via bronchoscopy.  Table 1 outlines this stepwise approach.

The American College of Chest Physicians (CHEST) guidelines advocate for a structured evaluation of pulmonary nodules in oncologic patients, integrating imaging with individual risk factors [24]. Nonetheless, as illustrated here, imaging alone is often insufficient. Comprehensive surveillance in RCC survivors must also consider opportunistic infections.

A bronchoscopy is still an important diagnostic tool when imaging is not conclusive. In this case, the bronchoscopy provided a definitive microbiologic diagnosis, thus avoiding unnecessary biopsy or empiric cancer-directed therapies. The Infectious Diseases Society of America (IDSA) guidelines state that bronchoalveolar lavage (BAL) is an important tool for diagnosing fungal infections in immunocompromised patients [25], such as those with CKD or who have received immune-modulating therapies related to nephrectomy.

Voriconazole remains the first-line therapy for CPA; however, its cyclodextrin vehicle may accumulate in patients with renal impairment, requiring careful dose adjustment [26, 27]. In our case, the antifungal therapy was well tolerated; however, monitoring renal function must be principal during treatment. Physicians following up patients (e.g., RCC survivors) with prior adverse renal outcomes must remain vigilant for possible harmful drug–drug interactions in the context of renal insufficiency.

In summary, this case report underscores the importance of comprehensive postnephrectomy RCC surveillance, extending beyond renal function monitoring to include vigilance for opportunistic infections arising from immune dysregulation, CKD, or environmental exposures. Early consideration of infectious mimics, particularly in RCC patients with persistent pulmonary nodules, can prevent unnecessary oncologic interventions and optimize patient outcomes.

## 4. Conclusion

A high index of suspicion for infectious mimics of malignancy can help avoid unnecessary invasive interventions and ensure timely, targeted treatment for diseases like pulmonary aspergillosis. With increasing awareness of immune dysregulation in patients with CKD and following nephrectomy, preemptive consideration is the key when infectious mimics of malignancy are suspected during workup of pulmonary nodules in RCC survivors.

## Figures and Tables

**Table 1:** Structured diagnostic approach to pulmonary nodules in a postnephrectomy RCC patient.

Clinical finding	Next step	Outcome
Pulmonary nodules in postnephrectomy RCC patient	Perform PET scan to assess metabolic activity	Proceed with PET evaluation
PET scan performed	Evaluate PET uptake and nodule characteristics	Classify as PET-avid (high malignancy risk) or nonavid (lower risk)
PET-avid nodules	Consider biopsy if high suspicion for malignancy	Possible metastatic RCC diagnosis ⟶ oncology referral
No significant PET uptake	Perform bronchoscopy and microbiologic testing	Alternative etiologies suspected ⟶ further testing required
Biopsy considered for malignancy	Analyze tissue for malignant cells	Definitive diagnosis of malignancy confirmed
Bronchoscopy & microbiologic testing	Obtain bronchoalveolar lavage (BAL) sample	Further analysis needed to differentiate infection
Histopathology confirms malignancy	Initiate oncology management	Cancer treatment initiated
Histopathology negative for malignancy	Expand differential (infection, granulomatous disease)	Reassess nodule etiology and management plan
BAL culture and staining performed	Check for fungal, AFB, and bacterial growth	Identify infection or rule out other causes
Positive for *Aspergillus fumigatus*	Start voriconazole or antifungal therapy	Aspergillosis diagnosis confirmed
Negative for fungal or bacterial infection	Consider other noninfectious etiologies	Consider other pulmonary conditions
Chronic pulmonary aspergillosis diagnosed	Monitor symptoms and repeat imaging	Long-term antifungal therapy considered
Empiric antifungal therapy started	Adjust antifungal based on response	Monitor renal function and adjust therapy
Clinical and imaging follow-up	Assess resolution or progression	Optimize management plan based on clinical course

*Note:* This table outlines the clinical decision-making process for evaluating pulmonary nodules in a postnephrectomy renal cell carcinoma (RCC) patient, differentiating between metastatic disease and infectious etiologies. The pathway begins with PET scan assessment, guiding further workup based on metabolic activity. PET-avid nodules warrant biopsy consideration, leading to either a confirmed malignancy diagnosis or the need for further evaluation. For non-PET-avid nodules, bronchoscopy with microbiologic testing is recommended to identify alternative etiologies, such as fungal or bacterial infections. A positive culture for *Aspergillus fumigatus* confirms chronic pulmonary aspergillosis, requiring antifungal therapy. This structured approach ensures accurate diagnosis, prevents unnecessary invasive procedures, and optimizes patient management in the context of RCC surveillance.

## Data Availability

The data that support the findings of this study are available from the corresponding author upon reasonable request.
